# Time-resolved X-ray spectroscopies of chemical systems: New perspectives

**DOI:** 10.1063/1.4953104

**Published:** 2016-05-31

**Authors:** Majed Chergui

**Affiliations:** Laboratoire de Spectroscopie Ultrarapide (LSU) and Lausanne Centre for Ultrafast Science (LACUS), ISIC-FSB, Ecole Polytechnique Fédérale de Lausanne, 1015 Lausanne, Switzerland

## Abstract

The past 3–5 years have witnessed a dramatic increase in the number of time-resolved X-ray spectroscopic studies, mainly driven by novel technical and methodological developments. The latter include (i) the high repetition rate optical pump/X-ray probe studies, which have greatly boosted the signal-to-noise ratio for picosecond (ps) X-ray absorption spectroscopy studies, while enabling ps X-ray emission spectroscopy (XES) at synchrotrons; (ii) the X-ray free electron lasers (XFELs) are a game changer and have allowed the first femtosecond (fs) XES and resonant inelastic X-ray scattering experiments to be carried out; (iii) XFELs are also opening the road to the development of non-linear X-ray methods. In this perspective, I will mainly focus on the most recent technical developments and briefly address some examples of scientific questions that have been addressed thanks to them. I will look at the novel opportunities in the horizon.

## INTRODUCTION

The field of time-resolved (picosecond and femtosecond) X-ray spectroscopies has witnessed an impressive growth over the past 10 years, thanks to the development of new methods and to the commissioning of new light sources. The field has been regularly reviewed in recent years,^1–8^ covering various aspects of the technical and scientific issues tackled by X-ray spectroscopy. However, the past 3 years have been rich in new developments both at synchrotrons and at X-ray Free Electron Lasers (XFELs).

For synchrotrons, the most important development has been the implementation^9^ of the high repetition rate scheme for optical pump/X-ray probe studies at synchrotrons. This not only brought about a significant increase in signal to noise (S/N) ratio for ps X-ray absorption spectroscopy (XAS) experiments,^9^ compared to the previous generation of 1 kHz experiment,^10–16^ but it also opened the door to ps X-ray emission studies, thanks to the sampling of weak emission signals.^17–19^ Finally, it has also been extended to time resolved X-ray photoelectron spectroscopy (XPS) studies.^20–24^

With the advent of XFELs, we have witnessed a significant improvement in time resolution (typical pulse widths of <100 fs), matched with a several orders of magnitude increase in a photon flux per pulse compared to synchrotrons, especially to the slicing scheme.^25–28^ These two aspects make XFELs a game changer for time-resolved X-ray spectroscopies. Aside from enabling fs XAS to be carried out with reasonable data acquisition times,^29^ they have made it possible to perform fs photon-in/photon-out experiments such as X-ray emission (XES)^30,31^ and resonant inelastic X-ray scattering (RIXS)^32,33^ studies, especially on dilute systems. It is important to stress that these latter classes of fs experiments can only be carried out at XFELs, making these instruments unique. The first short wavelength FEL to be built was FLASH at DESY (Hamburg, Germany) in 2005, which provided vacuum ultraviolet (VUV) and soft X-ray radiation.^34^ The first hard X-ray free electron laser Linac Coherent Light Source (LCLS) in the USA (Stanford) was launched in 2009, followed by the X-ray Ultraviolet (XUV)-FEL FERMI in Italy (Trieste)^35–40^ and the hard X–ray FEL SACLA in Japan,^41–46^ enabling many more studies to be carried on such instruments. In particular, the seeded FERMI XUV-FEL is the only true laser-like source and for that reason, it is blazing the trail to the implementation of non-linear X-ray optical techniques such as Four-wave mixing studies.^47–49^ In the following, we will briefly review the technical developments of the past 3–5 years and mention the new scientific problems that have been solved thanks to them. This will serve as a basis for discussing the new opportunities that are on the horizon both at synchrotrons and at XFELs.

## THE HIGH REPETITION RATE SCHEME

We previously reviewed the foregoing efforts aimed at exploiting nearly each X-ray pulses from synchrotrons, which usually run at MHz repetition rates, in order to increase the signal to noise ratio in time resolved X-ray experiments.^1^ After a number of earlier attempts that were not pursued,^20,50^ we implemented a high repetition rate scheme, with a specially designed data acquisition system at the Swiss Light Source (PSI Villigen), for studies of low concentration solution samples, especially proteins in physiological media. By using a ps high average power Nd:YLF laser (Duetto, TimeBandwidth Products) running at 520 kHz, while the hybrid pulses are delivered at 1.04 MHz at the Swiss Light Source (SLS), our group demonstrated an increase by a factor of ∼25 of the signal to noise ratio (S/N) per scan on a model spin cross-over (SCO) complex, [Fe(bpy)_3_]^2+^ in an aqueous solution with, as a consequence, greatly reduced data acquisition times and the ability to decrease the concentration of probed species to the mMol range.^9^ This development was followed by similar ones at the APS, where a high-repetition rate XAS setup was implemented at the 7-ID-D beam line, using the same pump laser as at the SLS.^51^ At Elettra (Trieste, Italy), a high-repetition rate scheme was implemented at the BACH beamline, which covers the energy range of 46–1600 eV with a full polarization control (linear horizontal and vertical, and circular clockwise and counter-clockwise).^21^ The laser sources used are a Coherent RegA9000 Ti:Sa amplified system that can operate at repetition rates ranging from 200 to 250 kHz with a pulse energy of 5 *μ*J and 100 fs pulse duration, or a Coherent Mira HP, which can operate at repetition rates of 0.01–5 MHz or at 83.3 MHz with a pulse energy of 25 nJ and a pulse duration of 100 fs at 800 or 400 nm. More recently, Gessner and co-workers have also implemented such a scheme at the ALS (Berkeley) for ps photoemission studies.^23,24^

These capabilities have allowed a certain number of experiments to be performed either with the much improved S/N compared to the previous generation of 1 kHz experiments,^52,53^ or importantly new experiments that could not be done before with the latter scheme.

### Picosecond XAS

**FIG. 1.**
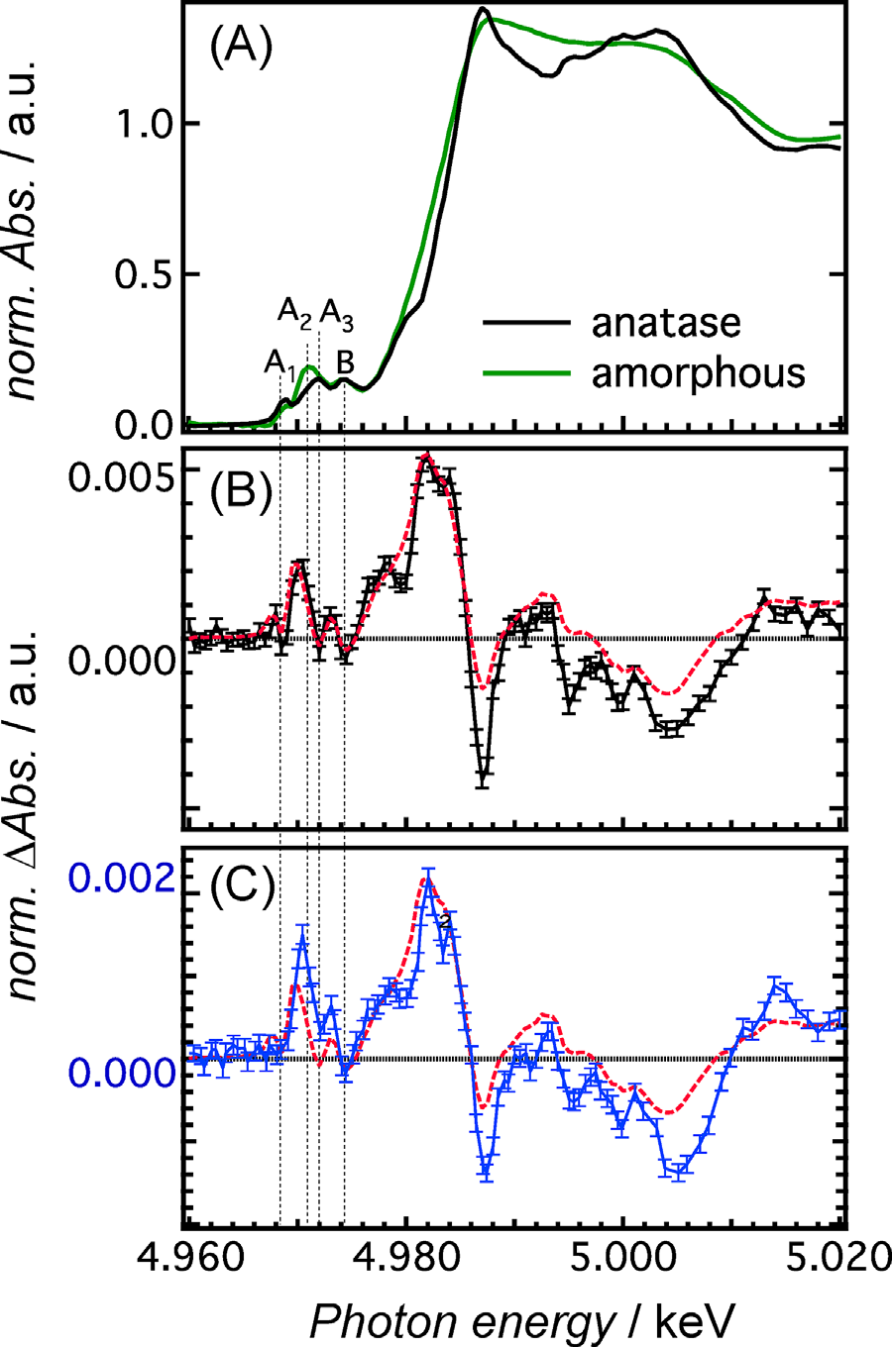
X-ray absorption spectra of colloidal solutions of anatase and amorphous TiO_2_ NPs (pre-edge and near-edge regions). (a) Ti K-edge absorption spectra of bare anatase and amorphous TiO_2_ NPs. The pre-edge peaks A_1_–A_3_ and B are due to dipole-forbidden 1s-3d transitions. (b) Transient X-ray absorption spectrum (XAS) of bare anatase TiO_2_ NPs excited at 355 nm at a time delay of 100 ps (black) together with the calculated difference spectrum (red dashed line). The latter is obtained by the amorphous steady-state spectrum shifted by ∼1 eV minus the anatase spectrum. (c) Transient XAS of the N719 ruthenium dye-sensitized anatase TiO_2_ NPs at a time delay of 100 ps (blue) after excitation at 532 nm. The dashed red trace is the same as in (b). Reproduced with permission from Rittmann-Frank *et al*., Ang. Chem. Int. Ed. **53**, 5858 (2014). Copyright 2014 Wiley-VCH Verlag GmbH & Co. KGaA, Weinheim.

A new series of measurements were performed on metal complexes such as rhenium,^54^ copper,^55^ and iron complexes^56^ in a solution, yielding clear signatures of the photoinduced electronic and structural changes. The technique was extended to the study of transition metal oxides, such as Titanium dioxide (TiO_2_) nanoparticles, both bare (under above band gap UV excitation) and sensitized by ruthenium dyes (under below band gap excitation).^57^ These latter experiment delivered a great insight into the nature of the Ti centred trapping sites where part of the electrons delivered to the conduction band (CB) end up. In particular, we could distinguish between the electron traps due to charge injection (at the outer surface) as opposed to those (deep inside the surface shell) transferred from the valence band by UV excitation (Figure 1). Ps XAS at high repetition rate studies have in the meantime been extended to other transition metal oxides, such as ZnO^58^ and WO_3_.^59^ Charge injection was also reported in the case of TiO_2_ sensitized by gold nanoparticles, but the laser and synchrotron sources were used as continuous sources and differences were taken between laser-on and laser-off RIXS spectra.^60^

**FIG. 2.**
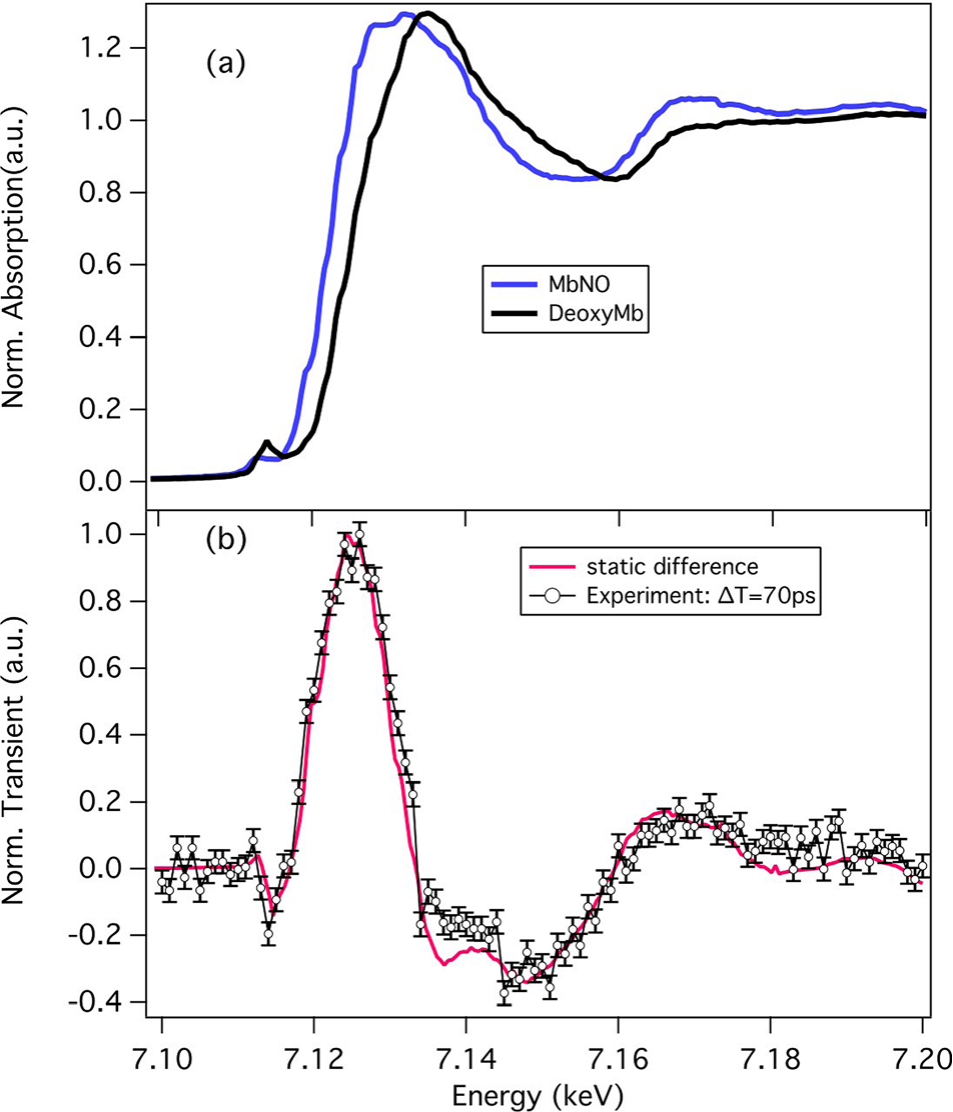
(a) Steady-state Fe K-edge absorption spectra of MbNO and (unligated) deoxy-Mb. (b) Transient spectrum (excited minus unexcited sample X-ray absorption) recorded 70 ps after excitation of the sample at 532 nm (o; with error bars). The difference of the deoxy-Mb minus MbNO (called the static difference spectrum) steady-state X-ray absorption spectra (a) is shown in red. The deviations around 7.135 keV and 7.165 keV between the two traces are due to the formation of a domed ligated heme. Reproduced with permission from Silatani *et al*., Proc. Natl. Acad. Sci. U.S.A. **112**, 12922 (2015). Copyright 2015 National Academy of Sciences.

One of our main aims in implementing the high repetition rate was to perform studies on low concentration samples such as proteins in physiological media.^9^ This goal was reached in a recent study of the NO ligand recombination to Myoglobin by ps Fe K-edge absorption (Figure 2),^61^ where we clearly established that the long-time component (∼200 ps) is due to diffusion of the NO ligand through the protein, during which the heme remains domed.

### Picosecond XES

As already mentioned, the high repetition rate scheme has enabled ps XES experiments to be performed routinely. While a first experiment had been performed using a 1 KHz set-up,^17^ its S/N was very poor. At high repetition rates, the quality of signals improves significantly^18,62,63^ and the recent studies on Fe-complexes are opening very promising prospects, especially when it comes to detecting the weak valence-to-core emissions, which contain information about the chemical bond.^19,64^ The Ps-XES studies have also been successfully demonstrated on photoexcited transition metal oxides such as ZnO.^58^

Because XES uses a fixed incident X-ray energy, it can simultaneously be combined with X-ray scattering, so that in addition to spin and electronic structure, one can probe the structural evolution of the system under study. This was nicely demonstrated by Bressler *et al*. in the ps time domain^19,62,63^ and has been extended to the fs time domain, as discussed below.

## X-RAY FREE ELECTRON LASERS

Until the advent of the XFEL, tuneable femtosecond X-ray pulses were only available at third-generation light source with a laser-electron slicing facility.^25–28,65^ This scheme was successfully used in the hard X-ray range to probe the ultrafast SCO in Fe(II) polypyridine complexes,^66^ and the changes of the solvent shell upon a photoinduced transition from a hydrophilic to a hydrophobic solvation.^67^ Using the slicing scheme with a UV pump, the first fs XAS experiments on transition metal oxides were carried out, showing that the CB electrons are trapped within 300 fs, reducing the Ti^4+^ ions to Ti^3+^.^68^ In the soft X-ray range, the slicing scheme was first used on materials probing the electronic rearrangements upon the photoinduced insulator-metal phase transition in VO_2_.^69^ Huse and co-workers implemented fs XAS of liquid solutions to investigate the SCO process in Fe(II)-polypyridine complexes at the Fe L-edges^70^ and the N K-edge.^71^

The hard X-ray FEL LCLS at SLAC (Stanford)^72,73^ generates 10^12^ hard X-ray photons per pulse at a repetition rate of 120 Hz, with a pulse duration <100 fs. Along with the SACLA facility (Japan),^74^ they have proved excellent sources for performing time-resolved X-ray experiments and single-shot structural studies.

Because of the inherent instability in both the photon energy, the pulse energy, and the pulse arrival time of these sources, which introduces a serious jitter, it was not straightforward to perform time-resolved XAS measurements^29^ with a resolution comparable to that of the slicing experiments, typically <200 fs.^66^ In the meantime, LCLS introduced a number of improvements, in terms of photon energy stability through a hard X-ray self-seeding^75^ to the timing jitter between the laser pump and X-ray probe pulses.^76,77^ The LCLS “timing tool,”^29,78^ which measures the timing jitter between the laser and the X-rays is such that the effective jitter is now ∼10 fs between optical and X-ray pulses, which is significantly shorter than the intrinsic uncorrected shot-to-shot time jitter (∼300 fs). At SACLA, Katayama *et al.*^79^ demonstrated the applicability of a beam branching scheme for photon diagnostics under a quasi non-invasive conditions. They used a transmission grating to generate multiple branches of X-ray beams. One of the two primary diffracted branches (+1st-order) was utilized for spectroscopy in a dispersive scheme, while the other (−1st-order) was dedicated for arrival timing diagnostics between the XFEL and the optical laser pulses. The transmitted X-ray beam (0th-order) was guided to an experimental station. They measured the correlation between the arrival timings of the −1st and 0th branches and found an error as small as 7.0 fs root-mean-square. These improvements notwithstanding, the main contribution to the overall time resolution in ultrafast X-ray spectroscopic measurements (typically ∼125 fs) at XFELs remain however the velocity mismatch of the optical and X-ray beams through the sample.^80^

The ability to now probe systems with a resolution of approximately 100 fs matched with the very high X-ray photon fluxes per pulse have led to an upsurge of several new experimental breakthroughs, which we briefly summarize here.

### Femtosecond XAS

We already mentioned the very first fs XAS experiment at an XFEL by Lemke *et al.*^29^ on an aqueous solution of [Fe(bpy)_3_]^2+^. More recently, and thanks to the above improvements in time resolution, Lemke *et al.*^81^ managed to resolve, for the first time, coherent wave packet oscillations induced by the impulsive spin cross-over transition, in agreement with the previous optical-only studies.^82,83^ This important achievement opens the door to similar highly insightful studies on a large class of molecular and biological systems. While the above two studies by Lemke *et al*. dealt with molecules in solution, fs XAS is meanwhile being extended to other classes of systems. In a recent study, Collet and co-workers reported fs XANES studies of a molecular crystals consisting of SCO molecules.^84^ In another study, Levantino *et al*.^80^ reported the first fs XAS experiment on a protein, Carboxymyoglobin (MbCO). However, they only recorded time traces using a fixed probe energy at the Fe K-edge, which makes the conclusions not straightforward. At SACLA, studies were performed on an Fe-complex, [Fe^III^(C_2_O_4_)_3_]^3+^ in solution,^85^ delivering insight into the photoinduced electronic and structural changes in the system, while the photoinduced charge carrier dynamics was investigated in the case of WO_3_ nanoparticles.^86^ These studies are widening the scope of systems one can investigate with fs XAS at XFELs. Finally, a dispersive XAS geometry^44,87,88^ has been proposed and tested as an alternative way to record fs XAS spectra in a single shot.

### Femtosecond photon-in/photon-out experiments

This class of femtosecond X-ray experiments, which includes XES and RIXS, can only be carried out at XFELs, and no wonder that it is with these methods that some of the most significant progress have been made at XFELs in the past 3 years.

**FIG. 3.**
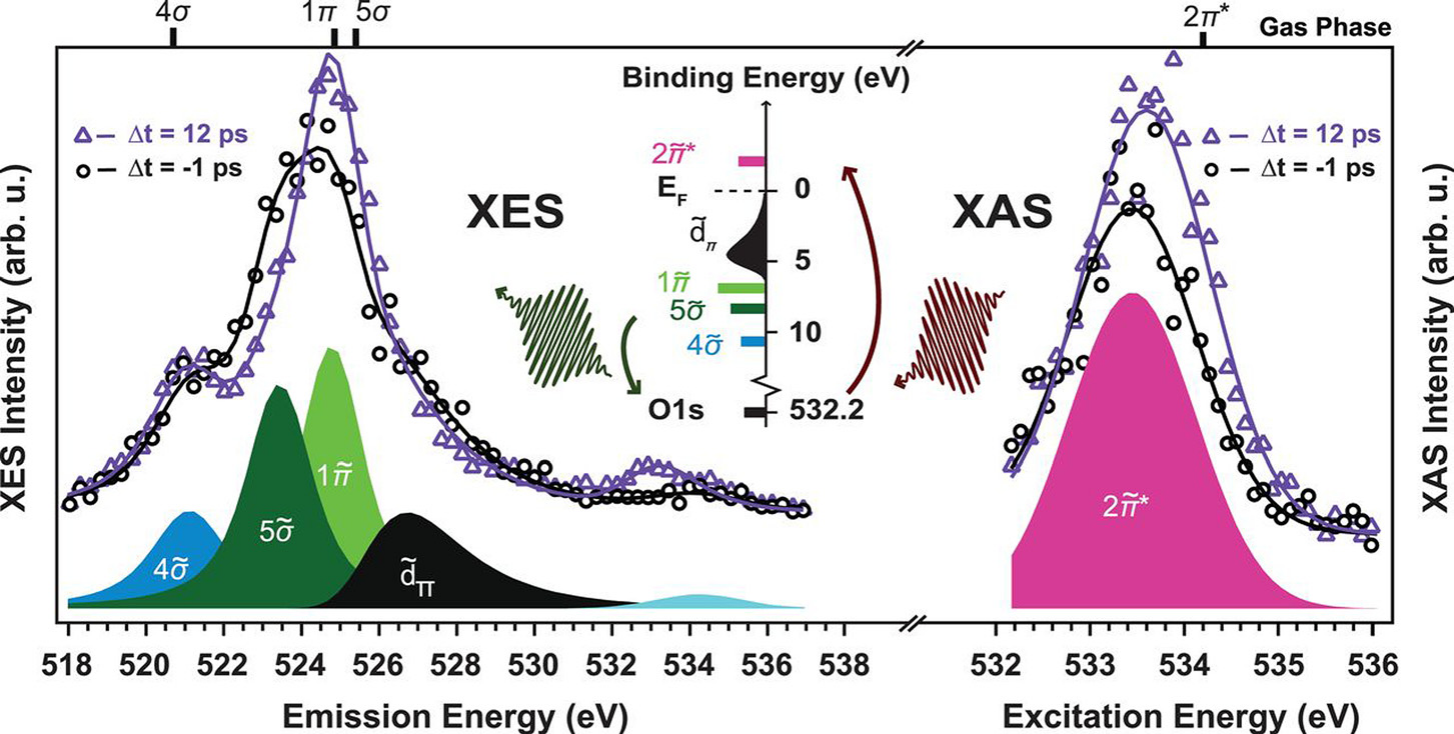
Oxygen K-edge XES (left) and XAS (right) spectra (markers) of CO/Ru(0001) and the corresponding fits (solid lines) measured at two selected pump-probe delays. The peak deconvolution resulting from the fit of the spectra acquired at Δt = −1 ps is shown with coloured lineshapes. The XAS data have been fitted with a Gaussian peak for the O1s→2$\tilde{\pi }$* resonance. The XES spectra have been fitted with three peaks of Voigt lineshape for the 1$\tilde{\pi }$, 5$\tilde{\sigma }$, and 4$\tilde{\sigma }$ orbitals and an asymmetric Gaussian for the d$\tilde{\pi }$ states; the elastic peak is indicated in light blue around 534 eV. The fit of the spectra at Δt = 12 ps has been performed by varying only intensity and position of the previously determined components. (Top) The positions of the fitted components measured in previous resonant gas phase experiments are also indicated.^89^ (Middle) A schematic illustration of the excitation process from the O1s level to the unoccupied 2$\tilde{\pi }$* resonance in XAS and the core hole decay process from occupied molecular orbitals back to the O1s in XES. Reproduced with permission from Dell'Angela *et al*., Science **339**, 1302 (2013). Copyright 2013 American Association for the Advancement of Science.

Nilsson and co-workers^89,90^ demonstrated ultrafast pump-probe x-ray fluorescence in the soft X-ray range at the LCLS. They used the X-ray pulses to probe the electronic structure of a transiently populated, weakly adsorbed state in CO desorption from Ru(0001). An optical laser pump pulse increased the phonon temperature of the substrate on a sub-picosecond time scale and rapidly populated the adsorbate transient state as an intermediate prior to desorption. The time evolution of the occupied and unoccupied valence electronic structure around the adsorbed CO molecule on Ru(0001) were followed in an element-specific way during the desorption process using oxygen resonant X-ray emission spectroscopy (RXES) and X-ray absorption spectroscopy (XAS), respectively (Figure 3). In particular, the CO molecules in the transient state had an electronic structure closer to the gas phase than to the chemisorbed state; however, the antibonding CO 2π* states were found to remain substantially be affected by the interaction with the surface. This first study was followed by a fs soft X-ray XAS experiment that investigated the formation of the CO_2_ molecule staring from CO and O on Ru(0001).^91^

**FIG. 4.**
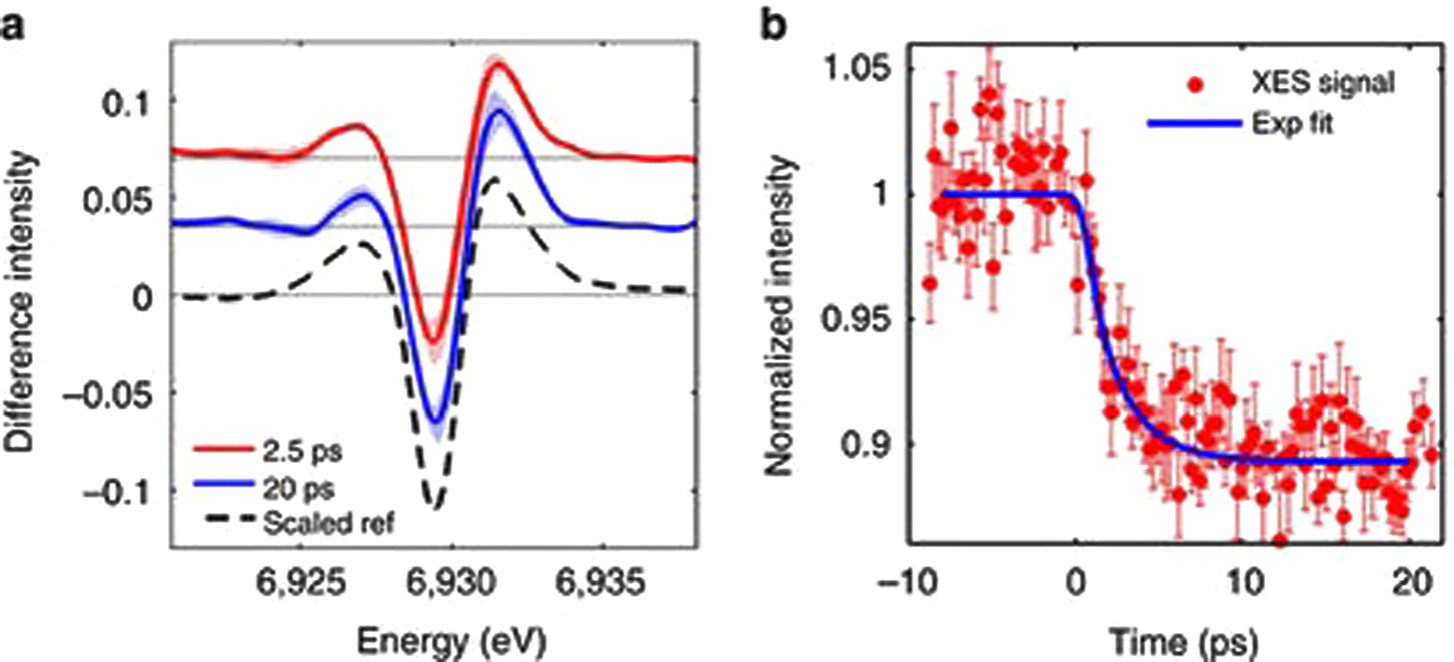
Ultrafast X-ray emission spectroscopy (XES) of the bimetallic RuCo complex [(bpy)_2_^1^Ru^II^(tpphz)^1^Co^III^(bpy)_2_]^5+^ (bpy = bipyridine, tpphz = tetrapyridophenazine): (a) Transient Co Kα1 at 2.5 (red) and 20 ps (blue) pump-probe delay. The shaded areas indicate the uncertainty level. The dashed black curve is the simulated reference for a ^1^Co^III^(LS)-^4^Co^II^(HS) conversion, scaled to the 20 ps trace. (b) Kinetic trace at 6.93 keV (red dots, with error bars) and single-exponential fit with a 1.9 ps lifetime, broadened by a 520 ± 410 fs XFEL Instrument response function (blue line). Reproduced with permission from Canton *et al*., Nat. Commun. **6**, 6359 (2015). Copyright 2015 Macmillan Publishers Limited.

More recently, Zhang *et al.* reported the first fs XES investigation of the spin cross-over (SCO) dynamics in photoexcited [Fe(bpy)_3_]^2+^.^30^ The idea of the experiment is to exploit the high sensitivity of XES to the spin state of the metal atom.^18,92–94^ The experiment was carried out with a resolution of <150 fs, which is not sufficient to fully resolve the SCO dynamics as recently shown in a laser-only pump-probe study at high time resolution (<50 fs).^83^ Nevertheless, the proof-of-principle of fs XES experiment is a very important achievement, which opens the way to entirely novel insights into photoinduced phenomena in chemical, biological systems and in materials. More recently, Canton *et al*.^31^ combined fs XES and X-ray scattering (XRS) in the hard X-ray range at the XFEL SACLA in Japan to characterize the non-equilibrated electron transfer (ET) dynamics in donor–acceptor molecular assemblies that contain optically dark active sites. The bimetallic complex they studied consists of a light-harvesting, ruthenium (Ru)-based chromophore linked to an optically dark cobalt (Co) electron sink by a bridge that mediates the ultrafast ET. This prototypical dyad exemplifies the wide class of synthetic and natural photocatalysts for which the coupled electronic and structural dynamics are only partially understood beyond the decay of the Franck–Condon state. They could thus nail down the dynamics of electron departure from the donor, the transit time via the bridge and the arrival to the acceptor, and finally, the formation time of the high spin state in the latter (Figure 4). The need for an observable such as XES is related to the fact that the Co-complex does not have known optical spectroscopic observables of its high spin state.

Very recently, Wernet and co-workers^32,33^ investigated the reaction dynamics of the transition-metal complex Fe(CO)_5_ in a solution, using fs RIXS in the soft X-ray range. The found that the photo-induced removal of CO generates the Fe(CO)_4_ species in a hitherto unreported excited singlet state that either converts to the triplet ground state or combines with a CO or solvent molecule to regenerate a penta-coordinated Fe species on a sub-picosecond timescale. This work stresses the relative importance of different spin channels in the photochemistry of Fe(CO)_5_. The advantage of using femtosecond X-ray spectroscopy is that it probes frontier-orbital interactions with atom specificity.

## NEW PERSPECTIVES

The above short review of the literature of the last three years shows that the stage is set for new opportunities to come. As far as time-resolved studies at synchrotrons are concerned, needless to say, that time-resolved X-ray spectroscopies at storage rings will be in high demand for several reasons: (a) phenomena on the 100 ps to several hundreds of ns (depending on the synchrotron) time scale will not require an XFEL, nor would it be wise to use the latter for such studies, given the high demand on these single user machines. There are a host of phenomena in biology, chemistry, and materials science that do not require a high temporal resolution, and these will best be studied at synchrotrons; (b) for many experiments to be planned at XFELs, it is highly advantageous to perform synchrotron-based experiments that establish observables and the level of signal, to be extrapolated to XFEL experiments; (c) as already mentioned above, synchrotrons will not only be useful for XAS but also for photo-in/photon-out experiments, as well as scattering and diffraction that do not require a sub-100 ps time resolution.^63^

As far as XFELs are concerned as invoked above, femtosecond photon-in/photon-out experiments can only be carried out at such machines, making them unique in this respect. The recent publications of several such studies^30,32,33,89,90^ are very promising and we can anticipate more of these studies, not only for molecular systems, but also for more complex systems such proteins, nano-systems, and materials. For proteins, XES is already very much used in the static mode,^95–98^ and extension to the time-resolved domain is only waiting for an opportunity. For materials, specifically single crystals, RIXS enables the Q-dependent study of collective excitations because a finite momentum can be transferred to the system from the X-ray photon.^99,100^ In a very recent work, Hill and co-workers reported the first fs magnetic RIXS after photo-doping the Mott insulator Sr_2_IrO_4_ and directly determined its magnetization dynamics. Although relevant to condensed matter physics, such studies are also of interest to chemical photovoltaics and photocatalysis when it comes to transition metal oxides.

There are additional avenues which we find promising at XFELs and are briefly presented below.

### High energy resolution off-resonant spectroscopy (HEROS)

One of the issues in recording the XAS spectra at an XFEL is that of the fluctuations in intensity and photon energy because XFELs operate on the SASE (self-amplified spontaneous emission) mode. This calls for both incident pulse energy normalization as well as incident spectrum normalization. The incident x-ray spectrum has been measured at the Linac Coherent Light Source (LCLS) on a pulse-to-pulse basis, but the spectrometer used is not broadly tunable in energy, making it only effective over a narrow photon energy range.^101^ The second drawback to this approach is the narrow bandwidth of the XFEL, approximately 0.25%–0.5%, restricting the range over which the XAS can be measured without requiring the incident photon energy to be scanned, something which is still non-trivial at the XFEL. However, recent experiments have demonstrated the feasibility of dispersive XAS spectroscopy in transmission mode at the SACLA XFEL (SPring-8, Japan).^44,87^

**FIG. 5.**
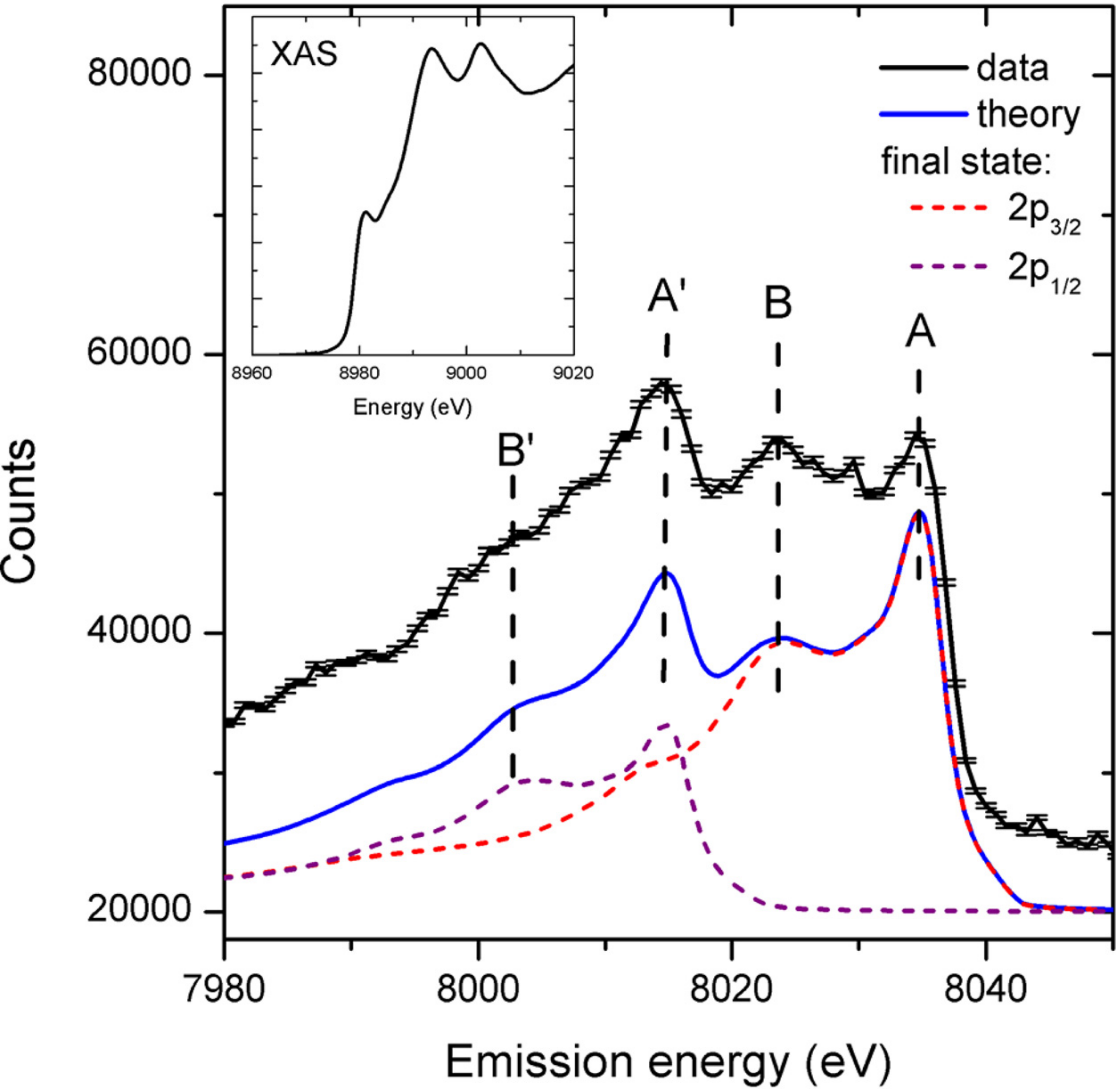
HEROS spectra of Cu metal for 2000 self-seeded shots (black curve). The error bars represent the standard deviation of the total counts. For comparison, we plot the calculated spectrum using the Kramers-Heisenberg relation and a Cu K-edge XAS spectrum recorded at a synchrotron facility shown in inset. The calculated curve represents the sum of two spectra relating to the final electronic states of 2p_3/2_ and 2p_1/2_. Reproduced with permission from Struct. Dyn. **1**, 021101 (2014). Copyright 2014 AIP Publishing LLC.

An alternative approach is the so-called High Energy Resolution Off-Resonant Spectroscopy (HEROS) method, which allows measurements of a scattered X-ray spectrum in a single acquisition that represents the unoccupied density of states. It is complementary to the ability of XES to probe the occupied density of states. HEROS can be used with a self-seeded XFEL beam to probe electronic states using single XFEL pulses. The idea of using off-resonant excitations is similar to pre-resonance Raman spectroscopy in the optical domain and likewise, it is described by the Kramers-Heisenberg relations.^102^ HEROS requires monochromatic photon energies for the incoming beam. One approach to generating this type of beam at an XFEL is the so-called “self-seeding” method,^103^ which allows the FEL to produce a narrow energy bandwidth beam with more stable beam characteristics than during normal SASE operation with a monochromator. Szlachetko *et al*.^104^ exploited this option at the LCLS and recorded the first HEROS spectrum using a single fs X-ray pulse. This offers a simple alternative to XAS and has the advantage that it can be combined with X-ray scattering experiments (Figure 5).

### Ultrafast non-linear X-ray optics

The high fluxes and coherence properties of XFELs are also opening the field to new non-linear X-ray spectroscopies, similar to what happened following the birth of the laser decades ago. Here, we briefly present some of the newest developments.

**FIG. 6.**
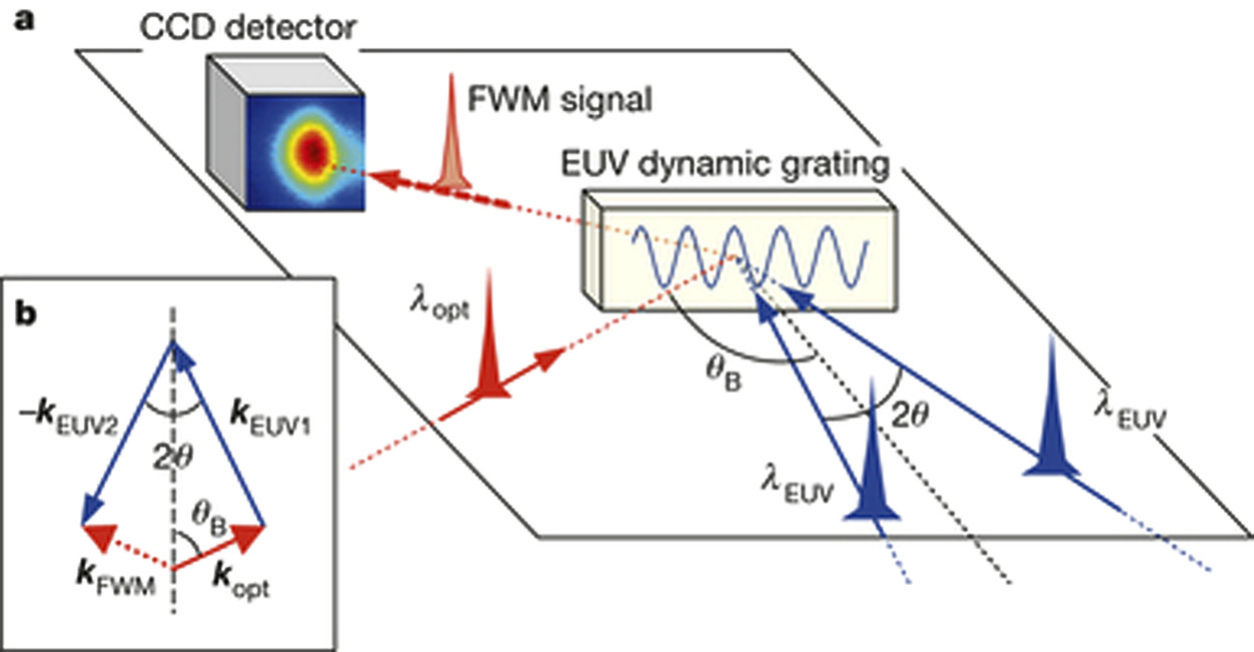
Four wave mixing (FWM) experiments with EUV transient gratings. (a) Sketch of the FEL-based FWM experiment: 2 EUV pulses (27.6 nm) and an optical pulse (392.8 nm) are crossed at well-defined angles. The two FEL beams generate the EUV dynamic grating on the sample. The time evolution of the TG is probed by the scattering of the optical pulse. A CCD sensor is placed in the expected propagation direction of the FWM signal beam, which is determined by the phase matching conditions of the three pulses. Reproduced with permission from Bencivenga *et al*., Nature **520**, 205 (2015). Copyright 2015 Macmillan Publishers Limited.

**FIG. 7.**
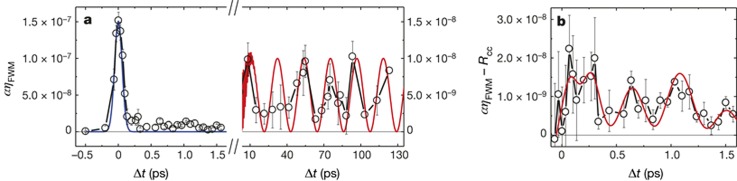
Time evolution of the EUV FWM signal of a vitreous SiO_2_ sample with the surface oriented orthogonally to the bisector of the FEL beams (Figure 4). (a) Black circles show the time dependence of the FWM signal, scaled to the intensity of the input beams. The blue and red lines are cross-correlation and the expected signal modulation (expanded for better visibility) due to acoustic modes, respectively. (b) Black circles connected by lines are the FWM signal after subtraction of the cross-correlation peak. The red line is the modulation due to oscillations of optical phonons at frequencies 1.15 THz and 4.1 THz. Reproduced with permission from Bencivenga *et al*., Nature **520**, 205 (2015). Copyright 2015 Macmillan Publishers Limited.

Four-wave mixing (FWM) processes, based on the third-order nonlinear light–matter interactions, can combine ultrafast time resolution with energy and wave vector selectivity, and enable the exploration of dynamics inaccessible by linear methods.^105,106^ The coherent and multi-wave nature of the FWM approach in the optical domain has been crucial in the development of advanced technologies. However, the use of optical wavelengths limits the spatial resolution and does not allow the probing of excitations with energy in the electron-volt range. Extension to shorter wavelengths—that is, the extreme ultraviolet and soft-X-ray ranges—would allow the spatial resolution to be improved and the excitation energy range to be expanded, as well as enabling elemental selectivity to be achieved by exploiting core resonances.^48,49,107–116^ So far, FWM at such wavelengths has been prevented by the absence of coherent sources of sufficient brightness and of suitable experimental set-ups. This is now possible and in a recent paper, Bencivenga *et al*.^113^ demonstrated how transient gratings, generated by the interference of coherent extreme ultraviolet pulses delivered by the FERMI free-electron laser can be used to stimulate FWM processes at sub-optical wavelengths. Figure 6 shows a sketch of the experiment: two extreme ultraviolet (EUV) FEL pulses (of a wavelength of 27.6 nm and estimated time duration 60–80 fs) are crossed at an angle 2θ onto a vitreous SiO_2_ sample with the surface oriented orthogonally to the bisector of the FEL beams. The interference of the two EUV pulses generates a transient grating with a spatial periodicity of 256.8 nm, which is probed using an optical pulse of 392.8 nm and 100 fs pulse width, coplanar with the FEL beams. The authors demonstrated the possibility of observing the time evolution of the FWM signal, which shows the dynamics of coherent excitations as molecular vibrations (Figure 7). This first non-linear EUV experiment opens the way for several new opportunities, such as two-colour EUV TG experiments with the two FEL pulses are resonant to core transitions of different atoms, or to an all-EUV TG experiment, where the probing pulse is also in the EUV and can be resonant with core-transitions of specific atoms. The next step is to achieve an all-EUV TG experiment, and the efforts are underway in this respect. This has the advantage of increasing the spatial resolution of the grating from *μ*m's in the optical domain to a few tens of nm's in the EUV.

The above short account about the developments in time-resolved X-ray spectroscopies over the past 3–4 years and the perspectives they offer is by no means exhaustive and several exciting possibilities are being explored at present, such as, e.g., (a) Adding a separate tuneable EUV source, in particular, a high harmonic generation (HHG) source,^117^ that would permit all-EUV non-linear optics experiments with a much enhanced degree of flexibility thanks to a larger choice of energies and phase matching conditions; (b) the idea of combining the high energy MeV electrons from the gun with X-ray pulses from the XFEL is another exciting option,^118^ particularly with the idea of using the X-ray as a pump to excite specific states of the system with atomic selectivity.

## CONCLUSIONS

As already mentioned, synchrotrons will go on playing an essential role in time resolved X-ray spectroscopic studies, both in absorption or in emission. The extension of time resolved XAS to the soft X-ray range in the regions of the L-edges (600–1000 eV) of transition metals is important, because of their role in coordination chemistry, photocatalysis, and biology. In this respect, the recent development of the flat jet technology^119^ is an important step forward as it allows samples that are thinner (1.5–4 *μ*m) than the penetration depth of the soft X-ray light.

The initial demonstrations of photo-in/photon-out experiments in the fs time domain at XFELs, along with the first implementations of ultrafast non-linear XUV experiments are promising great opportunities and let us envision the same evolution that has characterised optical lasers, e.g., new methods such as XUV multidimensional spectroscopies. It is also important to stress that these classes of experiment make the XFELs unique, as they can nowhere else be implemented. This is different to techniques, such as X-ray protein crystallography, whose practice is well proved and established at synchrotrons and keeps being improved.
